# Clinical and imaging correlates of amyloid deposition in dementia with Lewy bodies

**DOI:** 10.1002/mds.27403

**Published:** 2018-04-19

**Authors:** Paul C. Donaghy, Michael J Firbank, Alan J. Thomas, Jim Lloyd, George Petrides, Nicola Barnett, Kirsty Olsen, John T. O'Brien

**Affiliations:** ^1^ Institute for Ageing and Institute of Neuroscience Newcastle University Newcastle upon Tyne UK; ^2^ Nuclear Medicine Department Newcastle upon Tyne Hospitals National Health Service Foundation Trust, Newcastle upon Tyne UK; ^3^ Department of Psychiatry University of Cambridge Cambridge, UK

**Keywords:** Dementia with Lewy bodies, amyloid, positron emission tomography, florbetapir

## Abstract

**Background**: Amyloid deposition is common in dementia with Lewy bodies, but its pathophysiological significance is unclear.

**Objective**: The objective of this study was to investigate the relationship between amyloid deposition and clinical profile, gray matter volume, and brain perfusion in dementia with Lewy bodies.

**Methods**: Dementia with Lewy bodies (n = 37), Alzheimer's disease (n = 20), and controls (n = 20) underwent a thorough clinical assessment, 3T MRI, and early‐ and late‐phase ^18^F‐Florbetapir PET‐CT to assess cortical perfusion and amyloid deposition, respectively. Amyloid scans were visually categorized as positive or negative. Image analysis was carried out using statistical parametric mapping (SPM) 8.

**Results**: There were no significant differences between amyloid‐positive and amyloid‐negative dementia with Lewy bodies cases in age (*P* = .78), overall cognitive impairment (*P* = .83), level of functional impairment (*P* = .80), or any other clinical or cognitive scale. There were also no significant differences in hippocampal or gray matter volumes. However, amyloid‐positive dementia with Lewy bodies cases had lower medial temporal lobe perfusion (*P* = .03) than amyloid‐negative cases, although a combination of medial temporal lobe perfusion, hippocampal volume, and cognitive measures was unable to accurately predict amyloid status in dementia with Lewy bodies.

**Conclusions**: Amyloid deposition was not associated with differences in clinical or neuropsychological profiles in dementia with Lewy bodies, but was associated with imaging evidence of medial temporal lobe dysfunction. The presence of amyloid in dementia with Lewy bodies cannot be identified on the basis of clinical and other imaging features and will require direct assessment via PET imaging or CSF. © 2018 The Authors. Movement Disorders published by Wiley Periodicals, Inc. on behalf of International Parkinson and Movement Disorder Society.

Dementia with Lewy bodies (DLB) is the second most common type of neurodegenerative dementia. When compared with Alzheimer's disease (AD), it is associated with distinct cognitive, neuropsychiatric, and motor symptoms and characteristic imaging findings.^1^

The pathological hallmarks of DLB are the presence of Lewy bodies and Lewy neurites, but many cases also display AD pathology postmortem.^2^ However, the importance of concurrent AD pathology in DLB, at what point during the disease it occurs, and its relationships to clinical presentations are unclear.^3^ Positron emission tomography (PET) amyloid imaging allows the investigation of these relationships in vivo. In addition, as a result of the lipophilic nature of amyloid ligands such as Florbetapir, images collected immediately postinjection depict blood perfusion distribution.^4^ This allows comparison of amyloid deposition and perfusion without the need for an additional scan.

We examined amyloid deposition in patients with DLB who had detailed neuropsychological and clinical evaluations to assess the impact of amyloid on clinical profile. We hypothesized that in DLB, a positive amyloid PET scan would be associated with clinical and imaging findings similar to AD, that is, poorer memory function, hippocampal atrophy, and reduced medial temporal lobe perfusion. We also hypothesized that amyloid deposition in DLB would be intermediate between AD patients and healthy controls.

## Methods

### Participants

Participants were recruited prospectively between June 2013 and February 2016 from secondary care services in the North of England. Control participants were recruited through a research case register or were partners of participants. All participants were ≥60 years old. Dementia patients had a diagnosis of probable DLB or probable AD confirmed by 2 clinicians based on contemporaneous diagnostic criteria,^1^, ^5^ with an MMSE ≥12. The results of amyloid imaging were not known to the clinicians when making the diagnosis. Control participants had an MMSE ≥26 and no signs of dementia or mild cognitive impairment. DLB patients were recruited prior to the publication of the 2017 diagnostic criteria for DLB,^6^ but by definition, they would all satisfy the updated criteria for probable DLB.

Participants were excluded if they had a major concurrent psychiatric illness, severe physical illness, contraindications to PET‐CT imaging, a history of other significant neurological illness including stroke, previous experimental treatment with an amyloid‐targeting agent, or current treatment with any other investigational agent.

### Standard Protocol Approvals, Registrations, and Patient Consents

Participants with capacity gave their written informed consent to take part in the study. For those with dementia who lacked capacity, their participation in the study was discussed with a consultee in accordance with the Mental Capacity Act. The study received ethical approval from the National Research Ethics Service Committee North East—Newcastle & North Tyneside 2 (Research Ethics Committee Identification Number 13/NE/0064).

### Baseline Cognitive and Clinical Assessments

A thorough clinical assessment was carried out including measures of the following:

– Cognitive impairment (Addenbrooke's Cognitive Examination‐Revised,^7^ FAS Verbal Fluency,^8^ Trail Making Tests A and B,^9^ Graded Naming Test,^10^ Rey Auditory Verbal Learning Test,^11^ and computerized tests of simple and choice reaction times, digit vigilance, line angle discrimination, and motion detection^12^, ^13^, ^14^)
– Functional impairment (Bristol and Instrumental Activities of Daily Living Scales^15^, ^16^)
– Neuropsychiatric symptoms (Geriatric Depression Scale,^17^ Clinician Assessment of Fluctuations,^18^ Dementia Cognitive Fluctuations Scale,^19^ Neuropsychiatric Inventory^20^)
– Parkinsonism (revised UPDRS^21^).

### Imaging

Imaging was performed using a Siemens Biograph‐40 PET‐CT scanner (Siemens, Erlangen) in list mode. Participants were given a 370 MBq intravenous injection of ^18^F‐Florbetapir (Amyvid, Avid/Eli Lilly, Cork) followed immediately by a 5‐minute scan for perfusion images, with a 15‐minute scan (3 × 5 minute frames) starting 30 to 50 minutes after injection to image amyloid distribution. Images were reconstructed using iterative reconstruction (4 iterations, 16 subsets), with a 168 × 168 matrix size, 2.04 × 2.04mm pixel size, 3‐mm slice thickness, and 3‐mm postreconstruction Gaussian filter. Attenuation correction was performed using CT scan data.

CT scans were obtained immediately before the PET images. CT dose was minimized using the Siemens CARE Dose 4D protocol with 50 mAsec target dose, 0.5‐second gantry rotation time, 0.8‐mm beam pitch, 0.6‐mm slice thickness, and a scan duration of 19 seconds. Images were reconstructed with 3‐mm slice thickness to match the PET images. For participants without magnetic resonance imaging (MRI), CT scans were also reconstructed with a 1‐mm slice thickness for use as a background scan for visual rating.

MRI scans were performed on all patients unless contraindicated because of metal implants (n = 7). Scans were acquired on a 3T whole‐body MR scanner (Achieva scanner; Philips Medical Systems, Eindhoven), with body coil transmission and 8‐channel head coil receiver. Images acquired included a 3‐dimentional sagittal magnetization‐prepared rapid gradient echo sequence (repetition time 8.3 milliseconds, echo time 4.6 milliseconds, flip angle 8°, inversion delay 1250 milliseconds, imaging time 4.5 minutes). The sagittal acquisition matrix was 216 × 240, giving a voxel size of 1 × 1 × 1 mm.

### Image Processing

All analyses of MRI and PET images were performed using SPM 8 (http://www.fil.ion.ucl.ac.uk/spm/software/spm8/). A mean amyloid PET image was obtained by coregistering the 3 5‐minute scans undertaken 30 to 50 minutes postinjection. This mean scan was then coregistered with the native‐space MRI. MRI images were segmented into white matter, gray matter, and CSF. Gray and white matter images were smoothed using a 4‐mm, full‐width, half‐maximum Gaussian kernel to match the PET resolution. Region of interest maps were developed to mirror those used in the AD Neuroimaging Initiative^22^ using the MarsBar (http://www.marsbar.sourceforge.net) region map (Supplementary Table 1).^23^ In addition to the frontal, temporal, parietal, cingulate, and cerebellar areas used in the AD Neuroimaging Initiative, striatal and occipital regions were also included as imaging findings in these areas (occipital hypoperfusion and nigrostriatal denervation) are characteristic of DLB. These maps were transformed into native space for each participant. Cortical and white matter areas within these regions were identified using a threshold of 0.5 on the smoothed gray and white matter segmentation images. Mean PET uptake values within the regions of interest were then determined. Standardized uptake value ratios (SUVR) were calculated for each region by dividing the mean uptake in the region by the mean uptake in the cerebellum. The cerebellum has been the reference region of choice for cross‐sectional data in the AD Neuroimaging Initiative.^24^

A mean cortical SUVR was derived from the unweighted mean of the frontal, temporal, parietal, and cingulate regions.^22^

### Voxel‐Wise Analysis of Images

The mean amyloid PET image was coregistered with the perfusion PET image for each participant. The perfusion PET images were then spatially normalized to an age‐appropriate template,^25^ and the normalization parameters were applied to the amyloid images. Both the amyloid and perfusion images were smoothed with an 8‐mm Gaussian filter, and the images were intensity normalized using the mean of a cerebellum region of interest.^23^ This approach of normalizing via the perfusion scan was used because 7 participants did not have MRI images.

For the atrophy analysis, the segmented MRI images were processed using the Diffeomorphic Anatomical Registration Through Exponentiated Lie Algebra Toolbox to create a group‐specific template and spatially normalize images to it. The images were modulated to preserve the total tissue amount during normalization and smoothed with an 8‐mm Gaussian filter.

### Perfusion and Atrophy Region of Interest

In a previous paper investigating AD and DLB with perfusion single‐photon emission computed tomography (SPECT) and fluorodeoxyglucose (FDG) PET, we found that regions of interest in the parietal lobe (angular gyrus), medial occipital cortex, and medial temporal lobe had good specificity and sensitivity for distinguishing between dementia groups.^26^ We thus used the same regions to extract mean values from the normalized perfusion images. In addition, we determined the volume of the hippocampus from the gray matter segmentation using a previously described automated technique.^27^ Total gray matter, white matter, and CSF volume were also calculated from the segmentation images, and total intracranial volume is defined as the sum of these. Hippocampal volume and gray matter volume were analyzed relative to total intracranial volume.

### Visual Rating

Amyloid PET images were reviewed by 5 raters (J.O., M.F., G.P., J.L., and P.D.), all of whom had completed certified training in amyloid image reading. Raters were blinded to all clinical data. Scans were classified as positive or negative based on the method developed by the manufacturer.^28^ All scans were rated independently, following which a consensus meeting was held to discuss those scans in which there was disagreement (defined as a 3/2 split) on whether the scan was positive or negative, and a final group decision was reached.

### Statistical Analysis

Statistical analysis was completed using SPSS Statistics software (version 22; http://www-03.ibm.com/software/products/en/spss-statistics; IBM, New York). Normality was tested using the Shapiro‐Wilk test. Comparisons between diagnostic groups were carried out using one‐way analysis of variance or Kruskall‐Wallis tests with post hoc pairwise comparisons where significant (Bonferroni test α = .05). χ^2^ or Fisher's exact tests were used for categorical variables. Comparisons between amyloid‐positive and amyloid‐negative DLB cases were carried out using the general linear model and logistic regression; linear regression was used to investigate associations with mean cortical SUVR. Age and years in education were included as covariates for cognitive variables; age, sex, and years in education were included as covariates for imaging variables.

Voxel‐wise analyses were performed on the smoothed normalized images using the general linear model in SPM, with covariates of participant age and sex and (for MRI) total intracranial volume. Significant voxels (*P* < .001 uncorrected) were initially identified, followed by a cluster‐based family‐wise error correction (α = .05).

Participants with missing data were excluded from each analysis. The sample size was calculated to allow the detection of clinically useful differences between amyloid‐positive and amyloid‐negative DLB participants. It was anticipated that approximately 50% of DLB participants would be amyloid positive. A sample size of n = 15 to 20 per group would be sufficient to detect clinically useful differences (>1 SD) between the groups with 80% power.

## Results

### Comparison of DLB, AD, and Controls

A total of 87 volunteers agreed to enter the study, of which 77 met the eligibility criteria and completed their assessment. Supplementary Table 2 summarizes the group characteristics. The groups were well matched for age and sex, and the dementia groups were well matched for level of cognitive impairment.

DLB cases performed more poorly than controls on all cognitive tests (Supplementary Tables 2 and 3). The only difference between DLB and AD on cognitive testing was the digit vigilance task, with DLB cases displaying worse attention (digit vigilance number correct *P* = .01).

The proportion of DLB cases with a positive‐amyloid PET scan on visual rating was 20/37 (54%). This was significantly higher than controls (4/20 [20%]; *P* = .01), but significantly lower than AD (17/20 [85%]; *P* = .02).

Mean cortical amyloid deposition in DLB was intermediate between AD and controls, although it was not significantly different from either group (Table 1). DLB cases had higher amyloid deposition in occipital lobes when compared with controls (*P* = .04). Amyloid deposition was significantly higher in AD than controls in every cortical region, but there were no significant differences between AD and DLB cases in any region.

**Table 1:** ^18^F‐Florbetapir SUVR, cortical volume, and perfusion in DLB, AD, and control groups

Imaging Measure	Control	AD	DLB	*P*
Parietal SUVR, mean (SD)	1.10 (0.16)	1.37 (0.19)	1.25 (0.22)	.001^a^
Frontal SUVR, mean (SD)	1.11 (0.19)	1.38 (0.25)	1.25 (0.24)	.004^a^
Temporal SUVR, mean (SD)	1.10 (0.15)	1.36 (0.22)	1.25 (0.21)	.001^a^
Cingulate SUVR, mean (SD)	1.17 (0.21)	1.45 (0.25)	1.32 (0.25)	.003^a^
Occipital SUVR, mean (SD)	1.08 (0.12)	1.27 (0.18)	1.20 (0.18)	.002^a^, ^b^
Striatal SUVR, mean (SD)	1.11 (0.13)	1.28 (0.22)	1.19 (0.17)	.01^a^
Cortical SUVR, mean (SD)	1.12 (0.17)	1.39 (0.22)	1.26 (0.23)	.002^a^
Gray matter vol., mean (SD)	0.39 (0.03)	0.36 (0.03)	0.37 (0.03)	<.01^a^, ^b^
Hippocampal vol., mean (SD)	1.79 (0.20)	1.27 (0.26)	1.49 (0.36)	<.001^a^, ^b^^,^ c
Parietal perf., mean (SD)	1.04 (0.05)	0.87 (0.12)	0.86 (0.10)	<.001^a^, ^b^
MTL perf., mean (SD)	0.79 (0.05)	0.68 (0.08)	0.78 (0.09)	<.001^a^, ^c^
Occipital perf., mean (SD)	1.08 (0.05)	1.02 (0.08)	0.98 (0.07)	<.001^a^, ^b^
Occipital/MTL perf., mean (SD)	1.37 (0.07)	1.52 (0.18)	1.29 (0.17)	<.001^c^

One‐way analysis of variance or Kruskall‐Wallis tests with post‐hoc Bonferroni Correction (α = 0.05). Hippocampal and gray matter volumes are relative to total intracranial volume. SUVR measures: AD n = 19, DLB n = 31; volume measures: DLB = 31; otherwise control n = 20, AD n = 20, DLB n = 37. DLB, dementia with Lewy bodies; AD, Alzheimer's disease; SUVR, Standardized Uptake Value Ratio; vol., volume, perf., perfusion, MTL, medial temporal lobe.

a Significant difference control versus AD.

b Significant difference control versus DLB.

c Significant difference AD versus DLB.

SPM analysis of amyloid PET images found widespread amyloid deposition in AD when compared with controls, particularly in the frontal, temporal, and parietal areas (Supplementary Figure 1A). Deposition in DLB was less marked, but followed a similar distribution (Supplementary Figure 1B). There were few areas of higher deposition in AD when compared with DLB (Supplementary Figure 1C), none of which survived family‐wise error correction for significant clusters, although an occipital cluster approached significance (*P* = .052).

When compared with AD, the DLB group displayed higher hippocampal volume (*P* = .04), greater medial temporal lobe perfusion (*P* < .001), and a lower occipital:medial temporal lobe perfusion ratio (*P* < .001; Table 1).

On voxel‐wise analysis, when compared with controls, the DLB patients displayed temporoparietal atrophy and posterior hypoperfusion, with AD cases displaying more widespread atrophy and temporoparietal hypoperfusion (Supplementary Figure 2). DLB patients had less left medial temporal lobe atrophy, higher medial temporal and orbitofrontal perfusion, and lower perfusion in a part of the occipital cortex when compared with the AD patients (Supplementary Figure 3).

### Comparison of Amyloid‐Positive and Amyloid‐Negative DLB Patients

The amyloid‐positive and amyloid‐negative DLB groups were similar in age, although the amyloid‐positive group approached having significantly more years in education (*P* = .05; Table 2). There were no statistically significant differences in any clinical or cognitive test (Table 2, Supplementary Table 4). There was no difference between the 2 groups in duration of dementia (time from diagnosis to baseline assessment).

**Table 2:** Demographics and cognitive and clinical scales amyloid‐positive and amyloid‐negative dementia with Lewy bodies groups on visual rating

	Amyloid negative, n = 17	Amyloid positive, n = 20	*P*
Age, mean (SD), years	75.7 (5.6)	76.3 (7.3)	.78
Duration of dementia, mean (SD), months	25.9 (23.8)	20.5 (17.9)	.54
Sex, no. female (%)	3 (18)	3 (15)	1
Years in education, mean (SD)	10.3 (3.6)	12.6 (3.4)	.05
AChI/mem., no. (%)	16 (94)	20 (100)	.46
Antipsychotic, no. (%)	2 (12)	2 (10)	1
APOE ε4, no. (%)	8 (47)	13 (68)	.19
ACE‐R Total, mean (SD)	61.9 (13.3)	64.9 (16.5)	.83
ACE‐R Att./orient., mean (SD)	12.7 (3.6)	13.9 (3.2)	.52
ACE‐R Memory, mean (SD)	13.1 (4.8)	13.2 (6.0)	.36
ACE‐R Fluency, mean (SD)	5.2 (2.8)	6.8 (3.1)	.38
ACE‐R Language, mean (SD)	21.1 (3.4)	22.2 (2.8)	.95
ACE‐R Visuospatial, mean (SD)	9.9 (3.5)	8.9 (4.0)	.38
Rey Delayed Recall, mean (SD)	2.2 (2.8)	1.7 (2.4)	.22
Failed Trails A, no. (%)	8 (47)	6 (30)	.45
Failed Trails B, no. (%)	13 (77)	17 (85)	.24
FAS, mean (SD)	18.5 (12.2)	25.4 (12.9)	.44
GNT, mean (SD)	14.2 (7.8)	14.8 (8.3)	.46
IADL, mean (SD)	3.5 (2.3)	3.2 (2.0)	.75
BADL, mean (SD)	18.8 (12.9)	18.6 (13.2)	.80
NPI Hallucinations, mean (SD)	2.3 (2.4)	2.7 (2.8)	.92
NPI Hallucinations Distress, mean (SD)	0.8 (1.2)	0.8 (1.3)	.68
NPI Total, mean (SD)	15.9 (10.5)	24.1 (25.1)	.25
NPI Distress total, mean (SD)	7.2 (8.0)	10.2 (11.3)	.45
DCFS, mean (SD)	11.9 (4.0)	10.9 (3.8)	.50
CAF, mean (SD)	5.1 (4.2)	7.3 (4.5)	.07
GDS	5.1 (2.7)	4.5 (2.7)	.30
MDS‐UPDRS motor, mean (SD)	40.8 (16.5)	46.2 (18.4)	.58
Lying–standing sys. BP, mean (SD), mmHg	−16.1 (25.3)	−13.3 (24.1)	.30
Lying–standing dias. BP, mean (SD), mmHg	−1.3 (9.1)	−1.7 (10.4)	.64

General linear model for cognitive and clinical tests with continuous measures and logistic regression for dichotomous outcome variables with age and years in education as covariates. APOE ε4: amyloid positive n = 19; Rey Delayed Recall: amyloid positive n = 18; NPI Distress: amyloid positive n = 19; BP: amyloid negative n = 15, amyloid positive n = 18. AChI/mem., on acetylcholinesterase inhibitor or memantine; APOE ε4, ≥ 1 ε4 allele; ACE‐R, Addenbrooke's Cognitive Examination Revised; Att./orient, attention/orientation; FAS, FAS Verbal Fluency; GNT, Graded naming test; IADL, Instrumental Activities of Daily Living Scale; BADL, Bristol Activities of Daily Living Scale; NPI, Neuropsychiatric Inventory; DCFS, Dementia Cognitive Fluctuations Scale; CAF, Clinician Assessment of Fluctuation; GDS, Geriatric Depression Scale; MDS‐UPDRS, Unified Parkinson's Disease Rating Scale motor subscale; BP, blood pressure.

There were no differences between the amyloid‐positive and amyloid‐negative DLB groups in hippocampal volume or total gray matter volume (*P* > .20; Table 3). However, the amyloid positive group displayed lower medial temporal lobe perfusion (*P* = .03)

**Table 3:** Comparison of atrophy and perfusion between amyloid‐positive and amyloid‐negative dementia with Lewy bodies groups on visual rating

Imaging Measure	Amyloid negative	Amyloid positive	*P*
Gray matter volume, mean (SD)	0.37 (0.02)	0.37 (0.03)	.60
Hippocampal volume, mean (SD)	1.52 (0.25)	1.46 (0.45)	.55
Parietal perfusion, mean (SD)	0.87 (0.12)	0.85 (0.09)	.51
Occipital perfusion, mean (SD)	0.98 (0.08)	0.99 (0.05)	.93
MTL perfusion, mean (SD)	0.80 (0.05)	0.75 (0.10)	.03
Occipital:MTL perfusion, mean (SD)	1.23 (0.15)	1.33 (0.19)	.049

General linear model with age, sex, and years in education as covariates. Hippocampal and gray matter volumes are relative to total intracranial volume. Volume measures: amyloid negative n = 15, amyloid positive n = 16; perfusion measures: amyloid negative n = 17, amyloid positive n = 20. MTL, medial temporal lobe.

There were no differences between amyloid‐positive and amyloid‐negative DLB cases in atrophy or perfusion on voxel‐based analysis.

### Correlation of Semiquantification of Amyloid Deposition With Clinical and Imaging Findings in DLB

When entered into linear regression with age and years in education, there was no correlation between mean cortical SUVR and any symptom scale or cognitive test (*P* > .05) apart from an association with increased cortical SUVR and lower simple reaction time (β = −.57, *t* = −2.95, *P* < .01) and lower simple reaction time coefficient of variation (β = −.46, *t* = −2.14, *P* = .04). There was no significant correlation between mean cortical SUVR and Neuropsychiatric Inventory hallucinations, Clinician Assessment of Fluctuations, Dementia Cognitive Fluctuations Scale, or UPDRS total, gait, rigidity, rest tremor, or postural stability scores.

When entered into linear regression with age, sex, and years in education as independent variables, increased amyloid deposition was associated with lower medial temporal lobe perfusion (β = −.52, *t* = −3.07, *P* = .005) and a higher occipital:medial temporal lobe ratio (β = .65, *t* = 4.30, *P* < .001; Table 4). There was a trend toward an association with lower hippocampal volume (β = −.29, *t* = −1.87, *P* = .07).

**Table 4:** Relationship of mean cortical Florbetapir SUVR to cortical atrophy and perfusion

Imaging Measure	β (95% CI)	*P*
Hippocampal vol.	−0.29 (−0.60 to 0.03)	.07
Gray matter vol.	0.15 (−0.23 to 0.53)	.42
Parietal perf.	0.21 (−0.23 to 0.65)	.33
Occipital perf.	0.34 (−0.12 to 0.81)	.14
MTL perf.	−0.52 (−0.87 to −0.17)	.005
Occipital:MTL perf.	0.65 (0.34 to 0.96)	<.001

Linear regression with mean cortical Florbetapir SUVR, age, sex, and years in education included as independent variables. Hippocampal and gray matter volumes are relative to total intracranial volume. SUVR, Standardized Uptake Value Ratio; vol., volume; MTL, medial temporal lobe; perf., perfusion.

Striatal amyloid deposition has been linked to the severity of cognitive impairment in postmortem studies.^29^ We found that higher striatal SUVR was associated with lower simple reaction time (β = −.45, *t* = −2.2, *P* = .03). There were no significant associations between striatal SUVR and any other cognitive test or measure of functional impairment.

**Figure 1:**
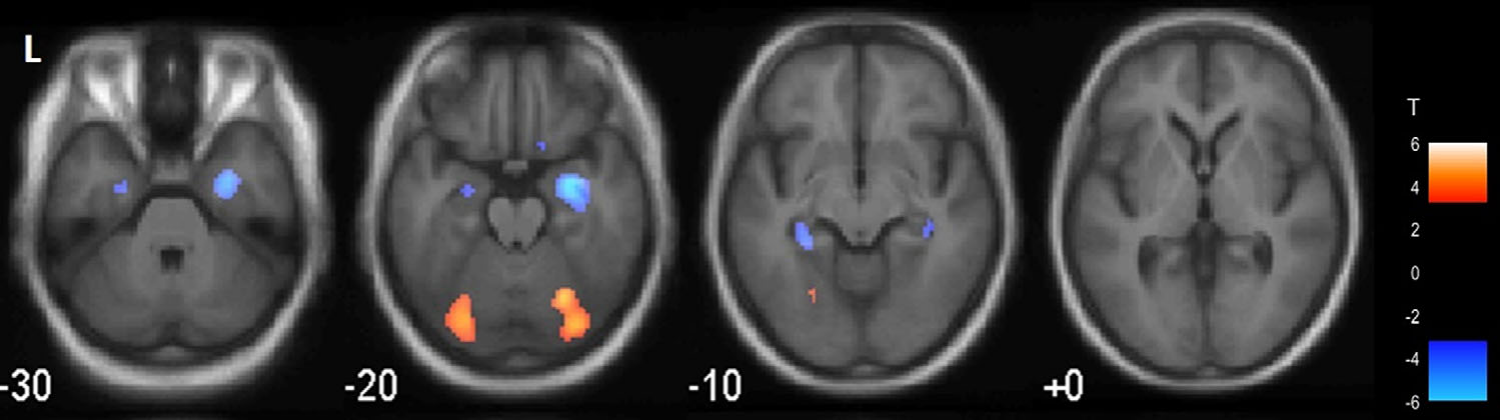
Voxel‐based correlation of amyloid deposition and perfusion in dementia with Lewy bodies. In uncorrected images (*P* < .001) there were 2 occipital clusters of positive correlation between amyloid deposition and perfusion and 2 hippocampal clusters of negative correlation. Following family‐wise error correction (not displayed), the right hippocampal cluster approached significance (*P* = .07). [Color figure can be viewed at http://wileyonlinelibrary.com]

Analysis of voxel‐based correlation between mean cortical amyloid deposition and gray matter density found no statistically significant clusters. However, there was an almost significant cluster of negative correlation between amyloid deposition and perfusion in the right hippocampus (*P* = .07, family‐wise error cluster‐wise corrected; Fig. 1).

### Identifying Amyloid‐Positive DLB Cases Without Amyloid Imaging

A post hoc discriminant analysis was carried out to test whether amyloid‐positive DLB cases could be identified without amyloid imaging. A simple model of age and medial temporal lobe perfusion yielded a sensitivity of 60% and specificity of 65% (Wilk's lambda *P* = .22) to identify amyloid‐positive cases. A model of age and occipital:medial temporal lobe perfusion gave a lower sensitivity (50%) but the same specificity (Wilk's lambda *P* = .18). A more complex model including age, medial temporal lobe perfusion, hippocampal volume, years in education, simple reaction time, and simple reaction time coefficient of variation yielded a sensitivity of 73% and a specificity of 64% (Wilk's lambda *P* = .07).

## Discussion

### Amyloid Deposition and Clinical Profile in DLB

Previous evidence, particularly from postmortem studies, has suggested that amyloid deposition in DLB may be associated with greater cognitive impairment,^30^ fewer core features of DLB,^31^ and greater brain atrophy.^32^ We hypothesized that amyloid‐positive DLB patients would have a clinical profile with similarities to AD, in particular greater memory impairment. However, we found no difference between amyloid‐positive and amyloid‐negative DLB cases on any cognitive test or measure of neuropsychiatric features, fluctuations, or parkinsonism. It is clear from our findings that it is not possible to identify the presence or absence of amyloid pathology in cases of probable DLB based on clinical profile. Direct measurement of amyloid through PET imaging or CSF measurement will be required for any research project wishing to identify DLB patients with concomitant amyloid deposition.

The lack of correlation between amyloid deposition and clinical profile may in part be because ^18^F‐Florbetapir binds to both neuritic and diffuse plaques. In contrast, pathological studies have reported associations between clinical profile and neuritic plaques only,^31^ or combined amyloid and neurofibrillary tangle pathology.^32^ Another factor may be that amyloid is an “upstream” factor in a cascade of events leading to neuronal damage and clinical symptoms.^33^ In this hypothesis, the severity of amyloid deposition would not reflect disease stage, but the presence of amyloid would be expected to be associated with more rapid disease progression. A recent study investigating CSF biomarkers in DLB found no baseline differences in cognition between those with an AD CSF profile and those with a normal profile.^34^ However, they did find that abnormal amyloid levels in CSF were associated with more rapid cognitive decline during follow‐up.

In contrast, tau pathology may be a “downstream” factor in this cascade and has previously been found to be less prevalent than amyloid pathology in DLB, but more closely related to clinical presentation.^35^ Early data from tau imaging studies have reported conflicting findings on the relationships between amyloid deposition, tau deposition, and clinical profile in DLB.^36^, ^37^ Other important pathophysiological factors such as the extent and severity of Lewy body pathology remain unquantifiable until postmortem.

### Amyloid Deposition and Imaging Variables in DLB

Amyloid‐positive DLB patients had lower medial temporal lobe perfusion, and there was a significant inverse correlation between mean cortical SUVR and medial temporal lobe perfusion. A trend toward an association between amyloid deposition and hypoperfusion was also observed in the right hippocampus on voxel‐based correlation. Two studies have reported negative findings when comparing amyloid‐positive and amyloid‐negative DLB cases with FDG uptake, although these studies had smaller sample sizes (n = 10^38^ and n = 21^39^).

There was no difference between amyloid‐positive and amyloid‐negative DLB patients in gray matter volume in region of interest and voxel‐based analyses. There was, however, a trend toward an inverse correlation between mean cortical Florbetapir SUVR and hippocampal volume. A recent longitudinal study found an association between baseline amyloid deposition and rates of decline in gray matter volume in posterior cingulate, temporal, medial temporal, and occipital regions, with the strongest association seen in the medial temporal lobe.^40^ As a result of the relatively subtle degree of atrophy observed in DLB, differences in atrophy between amyloid‐positive and amyloid‐negative patients may be difficult to detect in cross‐sectional analyses.

Our findings, along with the previous reported association between amyloid deposition and rates of brain atrophy, suggest that although there is little evidence of an association between amyloid deposition and an AD‐like clinical profile in DLB, amyloid is associated with imaging evidence of neurodegenerative changes in the brain, particularly in the medial temporal lobes. This could be because of the additive effects of amyloid pathology in DLB, the presence of tau pathology associated with amyloid, and/or synergistic interactions between amyloid/tau and α‐synuclein. MRI studies with postmortem confirmation have demonstrated a correlation between hippocampal atrophy and both amyloid and neurofibrillary tangle pathology.^41^, ^42^ Conversely, Lewy body pathology was found not to correlate with hippocampal volume,^42^ although α‐synuclein burden may decrease following cell death, making any such association difficult to detect.^43^ There is also evidence for synergistic interactions between AD pathology and α‐synuclein. Amyloid deposition has been associated with greater Lewy body pathology in Lewy body disease (Parkinson's disease and Lewy body dementia).^44^ Preclinical models have shown that the presence of tau promotes the fibrillization of α‐synuclein^45^ and that the presence of α‐synuclein increases the deposition of tau and amyloid in transgenic mice.^46^ Combined tau and amyloid imaging studies may help to clarify the contributions of each pathology to atrophy in DLB, although in the absence of an α‐synuclein imaging ligand the contribution of synergistic effects may be difficult to determine.

### Comparison of Amyloid Imaging Across Disease Groups

Consistent with previous research^47^ a positive amyloid PET scan on visual rating was more common in DLB than controls, but less common than in AD. On semiquantitative measurement, only the occipital lobes demonstrated significantly greater Florbetapir binding in DLB than controls, with no significant difference between DLB and AD or controls in any other region. However, in every region, DLB had binding values that were intermediate between controls and AD.

### Strengths and Limitations

This article presents the largest and most thoroughly profiled group of DLB cases to undergo amyloid imaging. We obtained a measure of brain perfusion through early Florbetapir imaging. This method has been shown to correlate with FDG‐PET imaging.^48^ The pattern of perfusion observed in this cohort was similar to that observed with hexamethylpropyleneamineoxime perfusion SPECT in a different cohort in this center.^26^

A total of 6 DLB patients and 1 AD patient were unable to have MRI scans as a result of metal implants. The same AD patient could only tolerate a 5‐minute amyloid PET scan. Their scan was not used in the quantitative analysis, but blinded to clinical diagnosis, and all 5 raters felt confident to visually rate the scan as positive. Some patients did not have complete data, principally as result of an inability to complete 1 or more parts of the cognitive assessment.

A total of 3 AD patients had negative amyloid PET scans. However, the proportion of cases with negative scans is in keeping with previous clinical samples in AD.^47^ A repeat analysis of clinical and cognitive comparisons was undertaken with the 3 amyloid‐negative AD patients removed from the dataset. The findings were unchanged. We also compared amyloid‐positive DLB and amyloid‐positive AD cases; there were no substantial differences to the findings of the overall comparison between DLB and AD.

We cannot exclude the possibility that the amyloid‐positive control cases reflect preclinical AD. Excluding these cases would increase the difference between DLB and controls, but the findings would be less generalizable to the general population.

Selection bias is an important factor to consider when interpreting these results. The DLB group in this cohort was typical of other cohorts in the literature and is reflective of cases of clinically diagnosed DLB. However, people with concomitant Lewy body and AD pathology who do not display the diagnostic symptoms of DLB will not be recruited into DLB groups in research studies. Thus, clinical samples of DLB may be more homogeneous in their clinical symptoms than pathological samples.

### Conclusion

We found no evidence for an association between amyloid deposition and clinical profile in this well‐characterized cohort. However, cortical amyloid deposition was associated with lower medial temporal lobe perfusion and a trend toward hippocampal atrophy. This may be a result of synergistic interactions between amyloid and α‐synuclein, additive effects of the 2 separate pathologies, or interactions between these proteins and other pathological proteins such as tau. As such, amyloid remains a potential treatment target in cases of DLB with substantial amyloid deposition. However, it will not be possible to identify such cases on clinical, cognitive, or other imaging grounds, and direct measurement of amyloid either through imaging or CSF analysis will be needed.

## Author Roles

1) Research project: A. Conception, B. Organization, C. Execution; 2) Statistical Analysis: A. Design, B. Execution, C. Review and Critique; 3) Manuscript: A. Writing of the first draft, B. Review and Critique.

P.C.D.: 1B, 1C, 2A, 2B, 3A

M.F.: 1A, 1B, 1C, 2A, 2B, 2C, 3B

A.J.T.: 1A, 1B, 2C, 3B

J.L.: 1B, 2C, 3B

G.P.: 1B, 2C, 3B

N.B.: 1B, 1C, 3B

K.O.: 1B, 1C, 3B

J.T.O.: 1A, 1B, 1C, 2A, 2C, 3B

## Full financial disclosures for the past 12 months

P.C.D. received funding from Lilly to attend a “Support for Physicians Involved in Research who are In Training” meeting. A.J.T. received support from GE Healthcare, the manufacturer of FP CIT (DaTSCAN), for investigator‐led research. G.P. received honoraria from GE Healthcare for delivering talks/training. J.T.O. has acted as a consultant for Lilly, has received research grant support from Avid/Lilly, and is supported by the National Institute for Health Research (NIHR) Cambridge Biomedical Research Centre. M.F., J.L., N.B., and K.O. report no disclosures.

## Supporting information

Additional Supporting Information may be found in the online version of this article at the publisher's website.

**Supplementary Table 1. Cortical regions of interest and corresponding MarsBar regions**

**Supplementary Table 2. Baseline demographic and cognitive test scores in DLB, AD and control groups**

**Supplementary Table 3. Computerised test scores in DLB, AD and control groups**

**Supplementary Table 4. Computerised test scores in amyloid positive and negative DLB**

**Supplementary Figure 1. Voxel‐based analysis of ^18^F‐Florbetapir binding in Alzheimer's disease (AD), Dementia with Lewy bodies (DLB) and control cases.** There are widespread areas of significant cortical deposition in AD compared with controls (A), with less widespread significant deposition in DLB, despite the greater number of subjects in this group (B). AD cases approached having significantly greater deposition than DLB in the occipital cluster illustrated (p=0.052).
AD = Alzheimer's disease; DLB = dementia with Lewy bodies. Voxelwise comparisons uncorrected at p=0.001 with Family‐Wise Error corrected clusters (α=0.05) except where stated.

**Supplementary Figure 2. Voxel‐wise comparisons of control subjects with dementia with Lewy bodies and Alzheimer's disease.** Compared with control cases, dementia with Lewy bodies displayed temporoparietal atrophy and posterior hypoperfusion. Alzheimer's disease cases displayed more widespread atrophy and temporoparietal hypoperfusion. Voxelwise comparisons uncorrected at p=0.001 with Family‐Wise Error corrected significant clusters (α=0.05).

**Supplementary Figure 3. Voxel‐wise comparisons between dementia with Lewy bodies and Alzheimer's disease.** When compared with Alzheimer's disease, dementia with Lewy bodies subjects had less left medial temporal lobe atrophy, higher medial temporal and orbitofrontal perfusion and a small area of lower perfusion in the occipital cortex. Voxelwise comparisons uncorrected at p=0.001 with Family‐Wise Error corrected significant clusters (α=0.05). Colour represents t statistic. Cross‐hairs are at MNI co‐ordinates [‐24,‐13,‐22].
