# Supplementary material for: Clinical and imaging correlates of amyloid deposition in dementia with Lewy bodies
Source: Mov Disord. 2018 Apr 19;33(7):1130–8. doi: 10.1002/mds.27403 (PMC6175485; doi:10.1002/mds.27403)
Supplement: Supplementary file 1 — Supplementary Table 1. Cortical regions of interest and corresponding MarsBar regions [file MDS-33-1130-s001.docx]

| **Supplementary Table 1. Cortical regions of interest and corresponding MarsBar regions** | |
| --- | --- |
| Region of interest | MarsBar Regions |
| Frontal lobe | Superior frontal gyrus  Middle frontal gyrus  Inferior frontal gyrus  Supplementary motor area  Paracentral lobule  Gyrus rectus  Olfactory cortex |
| Temporal lobe | Superior temporal gyrus  Herschel gyrus  Middle temporal gyrus  Inferior temporal gyrus |
| Parietal lobe | Superior parietal gyrus  Inferior parietal, but supramarginal and angular gyri  Angular gyrus  Supramarginal gyrus  Precuneus (middle and superior areas) |
| Cingulate lobe | Anterior cingulate and paracingulate gyri  Median cingulate and paracingulate gyri  Posterior cingulate gyrus  Precuneus (inferior area) |
| Occipital lobe | Superior occipital gyrus  Middle occipital gyrus  Inferior occipital gyrus  Cuneus  Calcarine fissure and surrounding cortex  Lingual gyrus  Fusiform gyrus |
|  | |
