# Supplementary material for: Clinical and imaging correlates of amyloid deposition in dementia with Lewy bodies
Source: Mov Disord. 2018 Apr 19;33(7):1130–8. doi: 10.1002/mds.27403 (PMC6175485; doi:10.1002/mds.27403)
Supplement: Supplementary file 2 — Supplementary Table 2. Baseline demographic and cognitive test scores in DLB, AD and control groups [file MDS-33-1130-s002.docx]

| **Supplementary Table 2. Baseline demographic and cognitive test scores in DLB, AD and control groups** | | | | |
| --- | --- | --- | --- | --- |
|  | Control (n=20) | AD (n=20) | DLB (n=37) | p |
| Age, mean (SD) | 75.9 (7.3) | 75.9 (6.8) | 76.0 (6.5) | 0.998 |
| Gender No. female (%) | 4 (20) | 4 (20) | 6 (16) | 0.91 |
| Years in Education , mean (SD) | 12.8 (2.9) | 11.5 (2.4) | 11.5 (3.6) | 0.15 |
| AChI/mem., No. (%) | 0 (0) | 20 (100) | 36 (97) | <0.001a,b |
| Antipsychotic No. (%) | 0 (0) | 0 (0) | 4 (11) | 0.10 |
| APOE ε4, No. (%) | 6 (30) | 15 (79) | 21 (58) | 0.01a |
| Time assessment to MRI, mean (SD), days | 17.4 (13.4) | 17.4 (9.9) | 29.9 (36.6) | 0.21 |
| Time assessment to PET, mean (SD), days | 28.5 (15.3) | 32.0 (22.7) | 28.5 (32.7) | 0.47 |
| ACE-R Total, mean (SD) | 94.8 (3.0) | 61.3 (17.2) | 63.5 (15.0) | <0.001a,b |
| ACE-R Att./Orient., mean (SD) | 18.0 (0.2) | 13.0 (3.7) | 13.4 (3.4) | <0.001a,b |
| ACE-R Memory, mean (SD) | 23.9 (2.0) | 10.0 (5.6) | 13.1 (5.4) | <0.001a,b |
| ACE-R Fluency, mean (SD) | 11.9 (1.6) | 6.3 (3.6) | 6.0 (3.0) | <0.001a,b |
| ACE-R Language, mean (SD) | 25.3 (0.9) | 21.0 (4.7) | 21.7 (3.1) | <0.001a,b |
| ACE-R Visuospatial, mean (SD) | 15.8 (0.6) | 11.3 (3.6) | 9.4 (3.8) | <0.001a,b |
| Rey Delayed Recall, mean (SD) | 8.6 (2.8) | 0.9 (2.0) | 1.9 (2.6) | <0.001a,b |
| Failed Trails A, No. (%) | 0 (0) | 4 (20) | 14 (38) | 0.005b |
| Failed Trails B, No. (%) | 0 (0) | 12 (60) | 30 (81) | <0.001a,b |
| FAS, mean (SD) | 49.7 (11.1) | 26.8 (13.1) | 22.2 (12.9) | <0.001a,b |
| GNT, mean (SD) | 24.2 (2.2) | 12.5 (7.0) | 14.5 (8.0) | <0.001a,b |
| IADL, mean (SD) | - | 4.3 (1.7) | 3.3 (2.1) | 0.09 |
| BADL, mean (SD) | - | 13.8 (9.8) | 18.7 (12.9) | 0.23 |
| NPI Hallucinations, mean (SD) | - | 0.2 (0.7) | 2.5 (2.6) | <0.001 |
| NPI Hallucinations Distress, mean (SD) | - | 0.2 (0.9) | 0.8 (1.2) | 0.01 |
| NPI Total, mean (SD) | - | 12.9 (10.8) | 20.3 (19.9) | 0.15 |
| NPI Distress Total, mean (SD) | - | 6.9 (7.9) | 8.8 (9.9) | 0.49 |
| DCFS, mean (SD) | - | 8.8 (4.0) | 11.3 (3.9) | 0.03 |
| CAF, mean (SD) | - | 4.2 (5.2) | 6.3 (4.4) | 0.046 |
| GDS | 2.1 (2.3) | 2.8 (2.9) | 4.8 (2.7) | <0.001b,c |
| MDS-UPDRS motor, mean (SD) | 5.6 (3.5) | 13.1 (6.2) | 43.7 (17.5) | <0.001b,c |
| Lying–stand sys. BP, mean (SD), mmHg | 3.9 (18.1) | 2.6 (22.7) | -14.6 (24.3) | 0.005b,c |
| Lying–stand dias. BP, mean (SD), mmHg | 7.6 (8.3) | 4.9 (9.4) | -1.5 (9.7) | 0.002b |
| a=significant difference control v AD, b=significant difference control v DLB, c=significant difference AD v DLB  Post hoc testing for each dependent variable carried out using Bonferroni Correction (α=0.05). DLB=dementia with Lewy bodies; AD=Alzheimer’s disease; AChI/mem.=on acetylcholinesterase inhibitor or memantine; APOE ε4= ≥1 Apolipoprotein ε4 allele; assessment=clinical assessment and cognitive testing; ACE-R=Addenbrooke’s Cognitive Examination Revised; Att./Orient=attention/orientation; FAS=FAS Verbal Fluency; GNT=Graded naming test; IADL=Instrumental Activities of Daily Living Scale; BADL=Bristol Activities of Daily Living Scale; NPI=Neuropsychiatric Inventory; DCFS=Dementia Cognitive Fluctuations Scale; CAF=Clinician Assessment of Fluctuation; GDS=Geriatric Depression Scale; MDS-UPDRS=Unified Parkinson’s Disease Rating Scale motor sub-scale; BP=Blood Pressure. (APOE ε4: AD n=19, DLB n=36; Rey Delayed Recall: AD n=18, DLB n=35; NPI Distress DLB n=36; DCFS: AD n=19; BP: DLB n=33) | | | | |
