# Supplementary material for: Clinical and imaging correlates of amyloid deposition in dementia with Lewy bodies
Source: Mov Disord. 2018 Apr 19;33(7):1130–8. doi: 10.1002/mds.27403 (PMC6175485; doi:10.1002/mds.27403)
Supplement: Supplementary file 3 — Supplementary Table 3. Computerised test scores in DLB, AD and control groups [file MDS-33-1130-s003.docx]

| **Supplementary Table 3. Computerised test scores in DLB, AD and control groups** | | | | |
| --- | --- | --- | --- | --- |
|  | Control (n=20) | AD (n=19) | DLB (n=34) | p |
| SRT, mean (SD), ms | 333.7 (51.0) | 454.4 (188.9) | 850.8 (1145.2) | <0.001a,b |
| SRT COV, mean (SD) | 0.22 (0.12) | 0.47 (036) | 0.49 (0.34) | <0.001a,b |
| CRT, mean (SD), ms | 555.9 (70.6) | 804.3 (379.7) | 1013.3 (459.0) | <0.001a,b |
| CRT COV, mean (SD) | 0.20 (0.06) | 0.33 (0.14) | 0.37 (0.18) | <0.001a,b |
| CRT errors, mean (SD) | 1.3 (1.7) | 5.4 (7.2) | 4.3 (4.1) | 0.004b |
| CRT-SRT, mean (SD), ms | 222.3 (58.2) | 350.0 (410.2) | 340.8 (365.7) | 0.015b |
| DV n identified, mean (SD) | 35.5 (0.9) | 29.7 (7.2) | 22.0 (8.9) | <0.001a,b,c |
| DV time, mean (SD), ms | 500.1 (60.2) | 603.9 (184.9) | 669.8 (191.3) | <0.001b |
| DV COV, mean (SD) | 0.13 (0.04) | 0.23 (0.12) | 0.30 (0.16) | <0.001a,b |
| Angle Task, mean (SD), ^o^ | 9.4 (3.7) | 33.0 (27.7) | 32.8 (28.0) | 0.001a,b |
| Motion Task, mean (SD) | 0.49 (0.30) | 0.79 (0.27) | 0.86 (0.23) | 0.002a,b |
| a=significant difference control v AD; b=significant difference control v DLB; c=significant difference AD v DLB  Post hoc testing for each dependent variable carried out using Bonferroni Correction (α=0.05)  DLB=dementia with Lewy bodies; AD=Alzheimer’s disease; SRT=Simple Reaction Time; COV=Coefficient of Variation; CRT=Choice Reaction Time; DV=Digit Vigilance.  (CRT: DLB n=33; DV: AD n=18, DLB n=30; Angle Task: Control n=17, AD n=17, DLB n=25; Motion Task: DLB n=22) | | | | |
