# Supplementary material for: Clinical and imaging correlates of amyloid deposition in dementia with Lewy bodies
Source: Mov Disord. 2018 Apr 19;33(7):1130–8. doi: 10.1002/mds.27403 (PMC6175485; doi:10.1002/mds.27403)
Supplement: Supplementary file 4 — Supplementary Table 4. Computerised test scores in amyloid positive and negative DLB [file MDS-33-1130-s004.docx]

| **Supplementary Table 4. Computerised test scores in amyloid positive and negative DLB** | | | |
| --- | --- | --- | --- |
|  | Amyloid negative (n=16) | Amyloid positive  (n=18) | p |
| SRT, mean (SD), ms | 888.5 (614.5) | 817.3 (1486.7) | 0.73 |
| SRT COV, mean (SD) | 0.61 (0.41) | 0.38 (0.23) | 0.07 |
| CRT, mean (SD), ms | 1157.6 (572.8) | 877.6 (271.1) | 0.27 |
| CRT COV, mean (SD) | 0.38 (0.17) | 0.37 (0.20) | 0.82 |
| CRT errors, mean (SD) | 4.2 (4.8) | 4.4 (3.5) | 0.67 |
| CRT-SRT, mean (SD), ms | 269.1 (453.4) | 408.4 (254.3) | 0.12 |
| DV n identified, , mean (SD) | 20.1 (10.6) | 23.3 (7.7) | 0.34 |
| DV time, mean (SD), ms | 657.0 (230.9) | 678.3 (166.6) | 0.49 |
| DV COV, mean (SD) | 0.31 (0.17) | 0.30 (0.15) | 0.79 |
| Angle task, mean (SD), ^o^ | 41.8 (32.7) | 25.8 (22.5) | 0.24 |
| Motion Task, mean (SD) | 0.89 (0.24) | 0.82 (0.22) | 0.39 |
| General linear model with age and years in education as covariates.  a=significant difference control v AD; b=significant difference control v DLB; c=significant difference AD v DLB  Post hoc testing carried out using Bonferroni Correction (α=0.05)  DLB=dementia with Lewy bodies; AD=Alzheimer’s disease; SRT=Simple Reaction Time; COV=Coefficient of Variation; CRT=Choice Reaction Time; DV=Digit Vigilance.  CRT: Amyloid positive n=17; Digit vigilance Amyloid negative n=12; Angle task: Amyloid negative n=11, Amyloid positive n=14; Motion Task: Amyloid negative n=10, Amyloid positive n=12) | | | |
