# Supplementary material for: Clinical and imaging correlates of amyloid deposition in dementia with Lewy bodies
Source: Mov Disord. 2018 Apr 19;33(7):1130–8. doi: 10.1002/mds.27403 (PMC6175485; doi:10.1002/mds.27403)
Supplement: Supplementary file 5 — Supplementary Figure 1. Voxel‐based analysis of 18F‐Florbetapir binding in Alzheimer's disease (AD), Dementia with Lewy bodies (DLB) and control cases. There are widespread areas of significant cortical deposition in AD compared with controls (A), with less widespread significant deposition in DLB, despite the greater number of subjects in this group (B). AD cases approached having significantly greater deposition than DLB in the occipital cluster illustrated (p=0.052). AD = Alzheimer's disease; DLB = dementia with Lewy bodies. Voxelwise comparisons uncorrected at p=0.001 with Family‐Wise Error corrected clusters (α=0.05) except where stated. [file MDS-33-1130-s005.docx]

| A B C 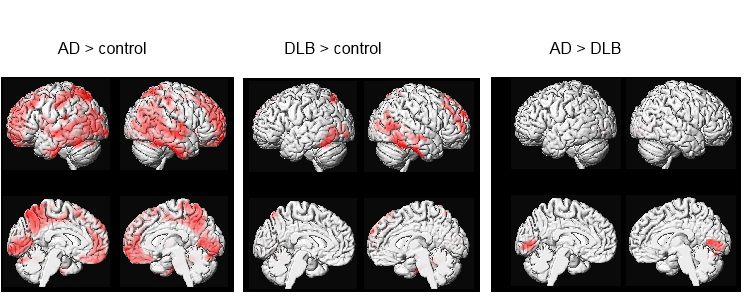 |
| --- |
| **Supplementary Figure 1. Voxel-based analysis of ^18^F-Florbetapir binding in Alzheimer’s disease (AD), Dementia with Lewy bodies (DLB) and control cases.** There are widespread areas of significant cortical deposition in AD compared with controls (A), with less widespread significant deposition in DLB, despite the greater number of subjects in this group (B). AD cases approached having significantly greater deposition than DLB in the occipital cluster illustrated (p=0.052).  AD = Alzheimer’s disease; DLB = dementia with Lewy bodies. Voxelwise comparisons uncorrected at p=0.001 with Family-Wise Error corrected clusters (α=0.05) except where stated. |
