# Supplementary material for: Clinical and imaging correlates of amyloid deposition in dementia with Lewy bodies
Source: Mov Disord. 2018 Apr 19;33(7):1130–8. doi: 10.1002/mds.27403 (PMC6175485; doi:10.1002/mds.27403)
Supplement: Supplementary file 6 — Supplementary Figure 2. Voxel‐wise comparisons of control subjects with dementia with Lewy bodies and Alzheimer's disease. Compared with control cases, dementia with Lewy bodies displayed temporoparietal atrophy and posterior hypoperfusion. Alzheimer's disease cases displayed more widespread atrophy and temporoparietal hypoperfusion. Voxelwise comparisons uncorrected at p=0.001 with Family‐Wise Error corrected significant clusters (α=0.05). [file MDS-33-1130-s006.docx]

| 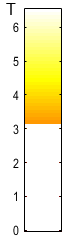Grey Matter Volume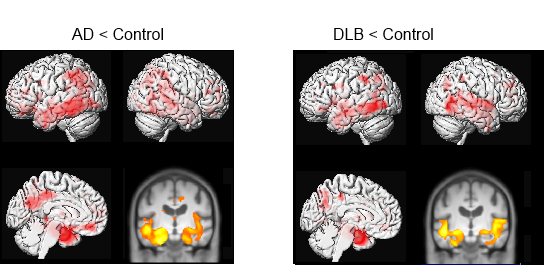  **L**  **L** |
| --- |
| Perfusion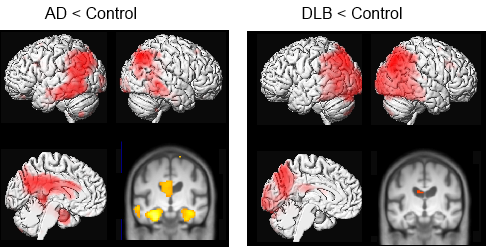  **L**  **L** |
| **Supplementary Figure 2. Voxel-wise comparisons of control subjects with dementia with Lewy bodies and Alzheimer’s disease.** Compared with control cases, dementia with Lewy bodies displayed temporoparietal atrophy and posterior hypoperfusion. Alzheimer’s disease cases displayed more widespread atrophy and temporoparietal hypoperfusion. Voxelwise comparisons uncorrected at p=0.001 with Family-Wise Error corrected significant clusters (α=0.05). |
