# Supplementary material for: Clinical and imaging correlates of amyloid deposition in dementia with Lewy bodies
Source: Mov Disord. 2018 Apr 19;33(7):1130–8. doi: 10.1002/mds.27403 (PMC6175485; doi:10.1002/mds.27403)
Supplement: Supplementary file 7 — Supplementary Figure 3. Voxel‐wise comparisons between dementia with Lewy bodies and Alzheimer's disease. When compared with Alzheimer's disease, dementia with Lewy bodies subjects had less left medial temporal lobe atrophy, higher medial temporal and orbitofrontal perfusion and a small area of lower perfusion in the occipital cortex. Voxelwise comparisons uncorrected at p=0.001 with Family‐Wise Error corrected significant clusters (α=0.05). Colour represents t statistic. Cross‐hairs are at MNI co‐ordinates [‐24,‐13,‐22]. [file MDS-33-1130-s007.docx]

| Grey matter volume: AD<DLB  **L**  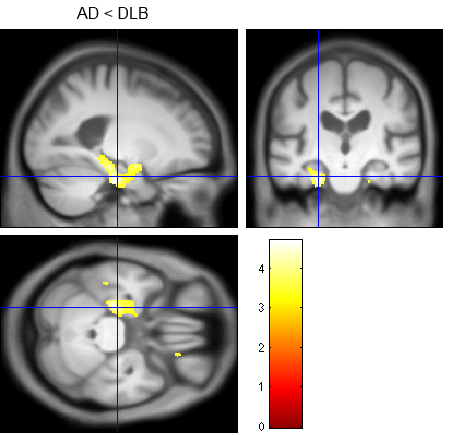  **L** | Perfusion: AD>DLB 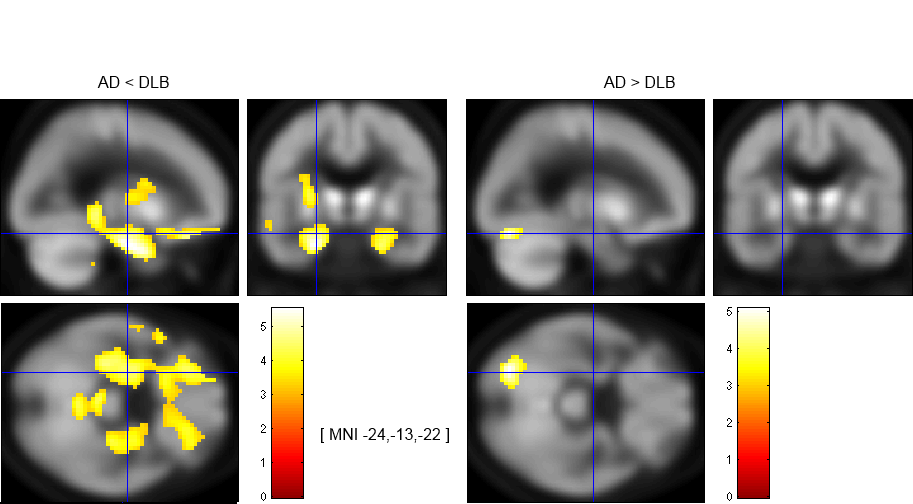  **L**  **L** | Perfusion: AD<DLB 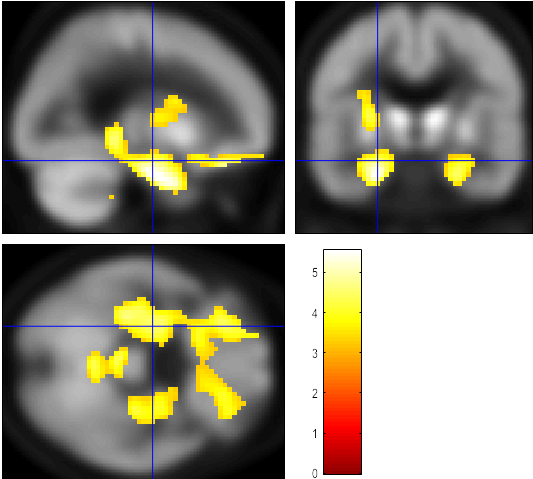  **L**  **L** |
| --- | --- | --- |
| **Supplementary Figure 3. Voxel-wise comparisons between dementia with Lewy bodies and Alzheimer’s disease.** When compared with Alzheimer’s disease, dementia with Lewy bodies subjects had less left medial temporal lobe atrophy, higher medial temporal and orbitofrontal perfusion and a small area of lower perfusion in the occipital cortex. Voxelwise comparisons uncorrected at p=0.001 with Family-Wise Error corrected significant clusters (α=0.05). Colour represents t statistic. Cross-hairs are at MNI co-ordinates  [-24,-13,-22]. | | |
